# Microbial Community Response to Terrestrially Derived Dissolved Organic Matter in the Coastal Arctic

**DOI:** 10.3389/fmicb.2017.01018

**Published:** 2017-06-09

**Authors:** Rachel E. Sipler, Colleen T. E. Kellogg, Tara L. Connelly, Quinn N. Roberts, Patricia L. Yager, Deborah A. Bronk

**Affiliations:** ^1^The Virginia Institute of Marine Science, College of William & Mary, Gloucester PointVA, United States; ^2^Department of Microbiology & Immunology, University of British Columbia, VancouverBC, Canada; ^3^Department of Marine Sciences, University of Georgia, AthensGA, United States

**Keywords:** dissolved organic matter, bacterial diversity, Arctic, Chukchi Sea, microbial community composition, tDOM

## Abstract

Warming at nearly twice the global rate, higher than average air temperatures are the new ‘normal’ for Arctic ecosystems. This rise in temperature has triggered hydrological and geochemical changes that increasingly release carbon-rich water into the coastal ocean via increased riverine discharge, coastal erosion, and the thawing of the semi-permanent permafrost ubiquitous in the region. To determine the biogeochemical impacts of terrestrially derived dissolved organic matter (tDOM) on marine ecosystems we compared the nutrient stocks and bacterial communities present under ice-covered and ice-free conditions, assessed the lability of Arctic tDOM to coastal microbial communities from the Chukchi Sea, and identified bacterial taxa that respond to rapid increases in tDOM. Once thought to be predominantly refractory, we found that ∼7% of dissolved organic carbon and ∼38% of dissolved organic nitrogen from tDOM was bioavailable to receiving marine microbial communities on short 4 – 6 day time scales. The addition of tDOM shifted bacterial community structure toward more copiotrophic taxa and away from more oligotrophic taxa. Although no single order was found to respond universally (positively or negatively) to the tDOM addition, this study identified 20 indicator species as possible sentinels for increased tDOM. These data suggest the true ecological impact of tDOM will be widespread across many bacterial taxa and that shifts in coastal microbial community composition should be anticipated.

## Introduction

As much as 50% of the world’s terrestrial organic carbon (C) pool is stored in the northern hemisphere as permafrost and approximately half of that is located within the upper 3 m (Gorham, 1991; Tarnocai et al., 2003, 2009). As atmospheric temperatures rise, the once semi-impermeable layers of permafrost begin to thaw, mobilizing a portion of this C pool by increasing the active layer depth, and then increasing riverine discharge and coastal erosion (Froese et al., 2008; Schuur et al., 2008; Feng et al., 2013; Holmes et al., 2013). This change in hydrology releases water, C, and nutrients, once trapped within the ice, into the coastal ocean. The amount of terrestrially derived dissolved organic matter (tDOM) supplied to the coastal ocean is hard to constrain due to complex environmental factors including changes in climate, hydrology and vegetation (Amon et al., 2012; Kicklighter et al., 2013). However, it is estimated that mobilization of organic C has increased by 3 – 6% from 1985 to 2004 (Feng et al., 2013) and will continue to increase with a warming climate (Amon et al., 2012). Bacterial production in the coastal Arctic appears to be controlled by the bottom up supply of C (Garneau et al., 2008) and the composition of C and nitrogen (N) pools can shape microbial community structure. These microbial responses can impact higher trophic levels, biogeochemical fluxes, and climate feedbacks (McCarren et al., 2010; Vincent, 2010). We hypothesize that tDOM increases will be important to the overall productivity of the Arctic Ocean and suggest that responsive microorganisms could be important sentinels of environmental change. Therefore, understanding what fraction of tDOM is bioavailable, how coastal microbial communities will respond to increases in tDOM and what impact these changes will have on microbial community composition and nutrient cycling is paramount.

Most of the freshwater runoff (both by water volume and DOM contribution) into the coastal Arctic Ocean happens during the short-window of freshet. The term freshet describes the pulse of freshwater that occurs in spring upon the melting of winter snow and ice accumulations. On the Alaskan Arctic coast, >50% of annual freshwater river discharge occurs within a 2 week period in spring (McClelland et al., 2014). This ephemeral fluvial discharge is accompanied by tremendous loads of tDOM and inorganic nutrients (McClelland et al., 2014). As a result, Arctic rivers are highly enriched in dissolved organic C (DOC) and N (DON) (Opsahl et al., 1999; Mathis et al., 2005; Holmes et al., 2008; Frey and Mcclelland, 2009), exposing coastal microbial communities to concentrated pulses of tDOM annually. The tDOM supplied by these Arctic rivers was once thought to be highly refractory (Opsahl et al., 1999; Dittmar and Kattner, 2003; Amon and Meon, 2004; Tank et al., 2012; Xie et al., 2012). Several studies, however, have found that a portion (10 – 40%) of this riverine tDOM is bioavailable on time scales of weeks to months (Hansell et al., 2004; Holmes et al., 2008; Alling et al., 2010; Mann et al., 2012; Vonk et al., 2013). This suggests that much of the bioavailable tDOM is being degraded within estuaries and the coastal ocean before it reaches off-shore waters.

Compared to more temperate regions of the world, study sites in the coastal Arctic are rare. Much of the existing research has focused on the major Arctic rivers, and comparatively little attention has been paid to the impacts of smaller rivers. This lack of knowledge impedes our ability to predict the impact of climate change on local Arctic food webs and biogeochemical cycles (Thingstad et al., 2008). In an effort to fill this knowledge gap, this study used tDOM from the Meade River, Alaska to address two main research objectives: (1) assess the bioavailability of tDOM to microbial communities in the coastal Chukchi Sea and (2) determine how pulsed, high concentration additions of tDOM, impact microbial community composition. These objectives were achieved through a series of bioassay experiments where extracted Meade River tDOM was supplied to microorganisms collected from coastal waters of the Chukchi Sea during ice-covered (April) and open-water (August) conditions. Biological [chlorophyll *a* (Chl *a*) and bacterial community composition] and chemical [ammonium (NH_4_^+^), nitrate (NO_3_^-^), DON, DOC, and phosphate (PO_4_^3-^)] changes were monitored throughout the experiments. This study provides the first evidence that some tDOM-responsive bacterial taxa could provide early indications of environmental changes in the coastal Arctic.

## Materials and Methods

### tDOM Collection and Extraction

Approximately 100 L of water was collected from a thermokarst draining into the Meade River, Alaska, at its mouth (70°54′39.1″N, 156°07′25.9″W), on 29 August 2010. The pH was 6.5, the salinity was 4 and the temperature was 7°C. The salinity of the samples is indicative of its proximity to the coast. The thermokarst water was collected into acid washed (10% HCl) high-density polyethylene (HDPE) carboys and transported in freeze safes (∼2 h transit) to the Barrow Arctic Research Center (BARC). The sample was filtered sequentially through pre-rinsed 5-μm polycarbonate filters, combusted (450°C for 4 h) Whatman Glass fiber filters (GF/F; nominal pore size 0.7 μm), and 0.2-μm Supor^®^ (PALL corp) filters. A 10% HCl solution was added to the filtered water to reduce its pH to 2. Samples were stored at 4°C until being shipped in freeze safes to the Virginia Institute of Marine Science (VIMS). Data loggers (HOBO TidbiT v2) were used to monitor water temperature at 60-s intervals during both the transit to BARC and VIMS. Sample temperatures did not increase more than 0.7°C during transit.

Two different extraction methods were compared to identify the method with the highest DOC recovery prior to the large-scale extraction efforts. Sub-samples of the source water were extracted using Superlite DAX-8 resin, described by Aiken (1985) for Amberlite XAD-8 resin or PPL solid phase extraction (Dittmar et al., 2008). Both extraction methods isolate and concentrate the DOM source retaining a fraction of the DOM pool but allowing salts (including inorganic nutrients) to pass through as filtrate. The DOC recovery using the DAX-8 resin was higher than the PPL extractions (61 and 48%, respectively) for the same aqueous sample. Because of its higher recovery, Superlite DAX-8 resin (Aiken, 1985) was used to extract the DOM used in this study. Once isolated, the salinity of the DAX extracted tDOM was adjusted to 30 using a combination of baked (500°C for 4 h) sodium chloride, magnesium sulfate and sodium bicarbonate. The change in salinity causes a portion of the tDOM to precipitate out of solution, mimicking the salinity induced flocculation that occurs within estuaries and deltas. After the salinity was adjusted, the higher salinity tDOM solution was re-filtered (0.2 μm) to remove any particulates that formed through this flocculation process, thus providing a more realistic geochemical signature of the tDOM. The proportion of total riverine DOM that is removed via salinity induced flocculation ranges between 3 and 11% (Sholkovitz, 1976). We found that ∼17% of the DOC was removed during the flocculation of the extracted tDOM (humic) fraction. Prior to use in the bioassay experiments, the tDOM source was brought to the *in situ* temperature of the experimental waters (-1°C or 4.5°C) to ensure additions of tDOM did not shock the receiving communities.

### Field Sample Collection and Bioassays

Seawater samples were collected from a site located 2.5 km northwest of Barrow, Alaska (71°20′39.6″N, 156°41′25.0″W). Whole (unfiltered) Chukchi Sea water was collected from 4 m depth in April and from a depth of 8 m in August. In April, the site was covered by 1.5 m of ice. The site was ice-free in August. Samples were collected in acid washed (10% HCl), 20 L HDPE carboys. The sampling procedures are described in detail in Baer et al. (2017). Bioassays were performed in April (26 April – 1 May 2011) and August (17 – 21 August 2011) and incubated in walk-in chambers located at the BARC in Barrow, Alaska. Temperatures were maintained at -1 °C in April and 4.5 °C in August, corresponding to ambient water temperatures at the time of collection. Both of these experiments were conducted under ambient light conditions, which was greater in August (∼50 μmol quanta m^-2^ s^-1^) than in April (4.7 μmol quanta^-1^ m^-2^ s^-1^), so both autotrophic and heterotrophic processes were apparent.

Pulsed DOM addition studies (Bioassays) are particularly applicable for the coastal Arctic. This is based on the well documented large pulses of high tDOM waters to the coast via Arctic rivers (Amon et al., 2012; Holmes et al., 2012, 2013; McClelland et al., 2014). While freshet represents the largest pulse of DOC to the coastal Arctic annually, the flow of DOM via rivers continues throughout the summer and can include pulses of DOM release (e.g., Holmes et al., 2008, 2012). Although DOC data in coastal waters during pulse events are limited, April DOC concentrations in the coastal Beaufort Sea range from ∼100 to 400 μmol C L^-1^ and August DOC concentrations range between ∼75 and 300 μmol C L^-1^ (Dunton and Crump, 2014). Our additions were 400 μmol C L^-1^ tDOM in both seasons. The same concentration and volume was used in April and August to reduce experimental variability and thus microbial response among the study seasons. Although 400 μmol C L^-1^ tDOM additions may be a bit high for summer incubations, with increasing atmospheric temperatures and the known C reserves contained within permafrost (Gorham, 1991; Tarnocai et al., 2003, 2009), the addition is realistic.

Bioassays were prepared by adding 1.25 L of substrate (extracted tDOM or artificial seawater in the Control treatments) to 8.75 L of whole seawater in acid washed (10% HCl), 10-L polycarbonate carboys, resulting in a 12.5% dilution of the ambient community. The addition of artificial seawater to the control ensured that the dilutions of the microbial community and ambient nutrient pools were consistent among both the control and tDOM addition treatments. This replication of dilution allowed us to specifically target the microbial response to the extracted tDOM. All bioassays were run in duplicate. Bioassays were sub-sampled for Chl *a*, bacterial community composition, and nutrient concentrations [DOC, total dissolved N (TDN; used to calculate DON), NO_3_^-^, nitrite (NO_2_^-^), NH_4_^+^, and PO_4_^3-^], at time points 0, 1, 2, 4, and 6 days in April and 0, 1.25, 2.5, and 4 days in August. The duration of the April incubation was longer (6 days) than the August incubation (4 days) due to a shorter field opportunity created by extensive weather delays in summer. Nutrient samples were filtered through combusted (450°C for 2 h) Whatman GF/F filters (nominal pore size 0.7 μm) and stored frozen until analyzed. Bacterial abundance was measured for ambient site samples in both April and August but bacterial abundance and production measurements within the bioassays were only made during the April sampling. The August bioassay bacterial abundance samples were collected but degraded prior to analysis and complications with shipping the radioisotopes to Barrow precluded production measurements in August.

### Nutrients and Dissolved Organic Matter Assessment

The colorimetric phenol-hypochlorite method was used to manually measure NH_4_^+^ concentrations in triplicate (Koroleff, 1983). Concentrations of NO_3_^-^, NO_2_^-^, and PO_4_^3-^ were measured using a Lachat QuikChem 8500 autoanalyzer (Parsons et al., 1984). Triplicate TDN and DOC concentrations were measured by high temperature combustion on a Shimadzu TOC-V TNM autoanalyzer (Hansell, 1993; Sharp et al., 2002, 2004). Deep-sea and low-carbon reference water from the University of Miami consensus reference material program were used as a quality control standard for DOC and TDN samples (Hansell, 1993). Concentrations of DON were determined as the difference between the TDN and dissolved inorganic N substrates; the errors from the TDN, NH_4_^+^, and NO_3_^-^ + NO_2_^-^ measurements were propagated to provide a standard error for DON.

Fourier transform ion cyclotron resonance mass spectrometry (FT-ICR MS) was used to assess the composition of the extracted tDOM. The extracted tDOM was analyzed in positive ionization mode using a hybrid 7 Tesla linear ion trap (LTQ) FT-ICR mass spectrometer equipped with an electrospray ionization (ESI) inlet system (LTQ FT Ultra, Thermo Electron Corp., Wood Hole Oceanographic Institute Mass Spectrometer Facility). Samples were analyzed in the positive ionization mode according to methods described in Sipler et al. (2013) and data were processed and internally calibrated according to Bhatia et al. (2010). FT-ICR MS data are reported as mass-to-charge ratios (m/zs), because the data presented here represent singly charged compounds; an individual *m/z* generally represents a single compound. The mass range analyzed was 100 – 1000 *m/z*. Compound classifications were assigned based on general hydrogen to C (H:C) and oxygen to C (O:C) molar ratios. General classifications and their corresponding ranges are described in Hockaday et al. (2009) and listed in **Table 1**. Multiple studies have used H:C and O:C molar ratios to relate the thousands of compounds generated using FT-ICR to more common compound classifications (e.g., Kim et al., 2003; Hockaday et al., 2009; Bhatia et al., 2010; Ohno et al., 2010; Sleighter et al., 2010; Sleighter and Hatcher, 2011). These classifications provide a general understanding of composition; however, the specific ranges and nomenclature used to distinguish among these classifications continues to evolve (e.g., Kim et al., 2003; Hockaday et al., 2009; Bhatia et al., 2010; Ohno et al., 2010; Sleighter and Hatcher, 2011). For example, the terms lignin, tannin and terrestrial have all been used to describe overlapping ranges (e.g., Hockaday et al., 2009; Bhatia et al., 2010; Killberg-Thoreson et al., 2013). Here we follow the ranges and nomenclature outlined by Hockaday et al. (2009). It should also be noted that although we have provided H:C and O:C ratios for each classification, a high degree of overlap among classifications is expected (Hockaday et al., 2009). We use these general classifications as a tool to group and visualize the potential composition and thus origins of the detected compounds. The aromaticity index (AI) was also calculated for all *m/z*s with molecular formula assignments (Koch and Dittmar, 2006; Šantl-Temkiv et al., 2013).

**Table 1:** Elemental ratios and compound classification characteristics of masses detected in extracted tDOM source using FT-ICR MS.

Classification	H:C range	O:C range	No. of m/z	% of Total
Lipid-like	1.5 to 2.0	0.0 to 0.3	494	9.2
Protein-like	1.5 to 2.2	0.3 to 0.67	673	12.6
Lignin-like	0.7 to 1.5	0.1 to 0.67	3049	57.1
Carbohydrate-like	1.5 to 2.4	0.67 to 1.2	143	2.7
Unsaturated Hydrocarbon-like	0.7 to 1.5	0.0 to 0.1	242	4.5
Condensed Aromatics	0.2 to 0.7	0.0 to 0.67	63	1.2
Other/unidentified	–	–	679	12.7


*Molecular formulas were assigned to 96% (5343 m/z) of the 5543 m/zs detected. Data represent the classification, range of H:C and O:C molar ratios, the number of *m/z*s per classification, and proportion of total *m/z*s detected in positive ionization mode using Fourier transform ion cyclotron resonance mass spectrometry (FT-ICR MS). Classifications described in more detail in Hockaday et al. (2009).*

### Biological Assessment

Concentrations of Chl *a* were measured fluorometrically (Parsons et al., 1984). Bacterial abundance was measured from triplicate samples taken from each incubation bottle and fixed with 4% formaldehyde. Abundance samples were stained with SYBR Green and counted on a flow cytometer (FACSCalibur, Becton-Dickinson). Fluorescing beads were used to approximate the number of cells mL^-1^ and at least 10,000 cells were counted per replicate. Bacterial production rates were measured via the incorporation of ^3^H-leucine into protein as a proxy for growth (Kirchman et al., 1985; Smith and Azam, 1992; Ducklow, 2000; Kirchman, 2001). Briefly, triplicate aliquots of 1.5 mL seawater from each treatment bottle were incubated in the dark in a water bath set to temperatures mimicking ambient temperatures for 4 h with 25 nmol L^-1^ of ^3^H-leucine (Williams et al., 2016). Incubations were stopped by rinsing each aliquot with 0.1 mL of 100% trichloroacetic (TCA). Protein was extracted by rinsing each aliquot with 1 mL of ice-cold 50% TCA and then by rinsing with 1 mL of 80% ethanol. Samples were centrifuged between each rinse. Parallel incubations for killed controls were done by adding ^3^H-leucine only after killing cells with 0.1 mL of 100% TCA. Activity measured in killed controls was subtracted from the sample values, eliminating any isotope adsorption to particulate protein.

### Bacterial Community Composition

Whole water from the sample sites and each incubation bottle was filtered onto a 0.2-μm Supor filter and frozen at -80 °C. Before extraction, 900 μL of DNA extraction buffer (Fortunato and Crump, 2011), lysozyme (2 mg/mL final concentration) and proteinase K (0.2 mg/mL final concentration) were added to the filters. Samples were then subjected to three freeze-thaw cycles. This was followed by enzymatic lysis in a 30 min 37°C incubation and then continued lysis at 65°C for 1 – 2 h after the addition of SDS (1% final concentration). Two phenol:chloroform:isoamyl alcohol (25:24:1) extractions were then carried out to isolate nucleic acids. Nucleic acids were then precipitated using 100% isopropanol (0.6 × volume of the resulting supernatant) for 2 h up to overnight, pelleted at 13,000 rpm for 30 min, and then rinsed and re-pelleted twice with 70% ethanol before being dried in a roto-evaporator. Once dry, samples were resuspended in 250 mL of nuclease-free water.

The V4 region (515F, GTGCCAGCMGCCGCGGTAA and 806R, GGACTACHVGGGTWTCTAAT) of the 16S rRNA gene for prokaryotic composition was amplified using Earth Microbiome Project protocols^1^, but with only 30 total cycles. This 806R primer has a bias against SAR11 sequences (Parada et al., 2015) and thus the relative abundance of these taxa and *Alphaproteobacteria* in general are likely underestimated in our study. Sample libraries were sent to Argonne National Lab for 2 × 150 bp sequencing on the Illumina MiSeq platform and reads were paired using *fastq-join* (Aronesty, 2013). Sequences that successfully joined were quality filtered, dereplicated (*derep_fulllength*) and abundance sorted (*sortbysize*) using UPARSE v 7.0.1001 (*fastq_filter)* (Edgar, 2013) with an expected error rate of 0.5. Singleton sequences were removed in the latter step in order to prevent them from seeding clusters when clustering operational taxonomic units (OTU). Reads were then clustered (*cluster_otus* in UPARSE pipeline) at 97% similarity. Reference-based chimera filtering was performed using UPARSE (*uchime*) with the Gold Database^2^ as the reference database. Reads (including singletons) were subsequently mapped back to OTUs using UPARSE (*usearch_global*) and an OTU table created. Taxonomy of the representative sequences was assigned in QIIME v 1.8 (*assign_taxonomy.py*; Caporaso et al., 2010) using the RDP classifier trained to the Greengenes database (v. 13.8^3^) for 16S amplicons. Any remaining singletons and OTUs occurring in only one sample, chloroplast, mitochondrial and archaeal sequences were removed in QIIME (*filter_otus_from_otu_table.py*). Sequences passing these quality control filters were uploaded to the NCBI Short Read Archive (SAMN06175504 to SAMN06175533) under the BioProject PRJNA310254. Samples were subsampled to 3037 sequences per sample (the minimum number of sequences per sample in the dataset) before subsequent analyses.

### Statistical Analyses

We used ANOVAs (Nelson et al., 2013) and Tukey’s HSB test, which controls for multiple comparisons, to determine if an OTU significantly changed (*p* < 0.05) over the course of the bioassay (among any of the treatments) and the treatments and time points between which these OTUs changed. These analyses were only run on OTUs that were present in four or more samples and were abundant or became abundant during at least one of the time points. Here we define an abundant OTU as an OTU that reached > 1% of the sample at some point over the course of the bioassay. Replicates were collected from each sample bottle and were grouped by treatment and time point for these statistical analyses. We then calculated the fold-change between the relative abundance of these OTUs at 4 days and in the starting community to determine the magnitude of change in the relative abundance of these significant OTUs over the course of the experiments. Relationships between the average relative abundance of the OTUs that changed throughout the bioassays and average nutrient, Chl *a* and organic matter concentrations over the course of the bioassays were assessed using Spearman rank correlations. Non-metric multidimensional scaling (MDS) plots were computed in R, using metaMDS in the VEGAN package (Oksanen et al., 2016) and the stress for this ordination (0.0362) was calculated using nmds.monte in the BIOSTATS collection of R functions (Kevin McGarigal^4^).

## Results

### Site Characteristics

**FIGURE 1:**
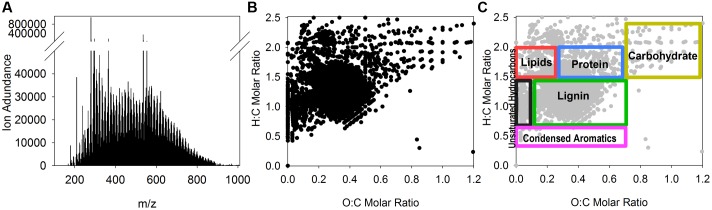
Compound level evaluation of extracted tDOM. Fourier transform ion cyclotron resonance mass spectrometer was used to evaluate the composition of the extracted tDOM. The presented data include **(A)** FT-ICR mass spectra of extracted tDOM source showing the relative ion abundances of detected mass-to-charge ratios (*m/z*s); **(B)** Van Krevelen diagram showing the hydrogen to carbon (H:C) and oxygen to carbon (O:C) molar ratio distributions of masses for which molecular formulas could be assigned (96% of detected *m/z*s); **(C)** Van Krevelen diagram of the same points shown in **(B)** overlaid with general compound class ranges described in Hockaday et al., 2009).

The thermokarst water sample contained no detectable concentrations of NH_4_^+^ or NO_3_^-^, but was highly enriched (relative to seawater) in DOC and DON (**Table 2**). Sixty-one percent of the thermokarst DOC and 56% of the DON pools were retained during tDOM isolation. The molar DOC:DON ratio within the thermokarst water and extracted tDOM were 24 and 26, respectively. Molecular formulas were assigned to 96% (5343 m/z) of the 5543 m/zs detected in the extracted tDOM sample by FT-ICR MS (**Figure 1**). Masses were detected across the entire analytical range (100–1000 Da.) but the average molecular weight of compounds detected in this sample was 590 Da. The average H:C of detected *m/z*s was 1.39 and the average O:C of detected *m/z*s was 0.33 which is similar to other lignin and tannin-rich natural DOM samples from temperate swamps (e.g., Sleighter and Hatcher, 2008; Hockaday et al., 2009; Ohno et al., 2010) and other Arctic permafrost thaw streams (Spencer et al., 2015). Based on the H:C and O:C molar ratios of the individual *m/z*s, the tDOM was dominated by lignin-like compounds which accounted for 57% of the *m/z*s for which molecular formulas could be assigned (**Table 1**). Of the remaining masses, protein, lipid and unsaturated hydrocarbon-like compounds represented 13, 9, and 5% of the compound pool, respectively. Approximately 13% of compounds for which molecular formulas were assigned could not be categorized under the general H:C and O:C molar ratios for known classifications (**Table 1**). For comparison, we also calculated the AI of the compounds for which molecular formulas could be assigned (Koch and Dittmar, 2006; Šantl-Temkiv et al., 2013). Using this approach we found that approximately 37% (1960 *m/z*s) of compounds could be categorized as moderately unsaturated, and aromatic and condensed aromatic each accounted for 16 and 15%, respectively. Approximately 64% of compounds that were identified as lignin-like were also identified as moderately unsaturated showing similarities between these two distinctions.

**Table 2:** Biological and chemical concentrations in the tDOM source and ambient site water.

	Chl *a*μg L^-1^	Bacterial Abundance 10^8^ cells L^-1^	Ammonium μmol N L^-1^	Nitrate μmol N L^-1^	DON μmol N L^-1^	Phosphate μmol P L^-1^	DOC μmol C L^-1^
Thermokarst	ND	ND	BD	BD	155 ± 0	0.36 ± 0.01	3670 ± 17
Extracted tDOM	ND	ND	0.80 ± 0.52	BD	600 ± 14	0.08 ± 0.01	15430 ± 31
April	0.10 ± 0.01	2.58 ± 0.10	0.81 ± 0.05	8.41 ± 0.01	4 ± 0	1.05 ± 0.01	68 ± 1
August	0.37 ± 0.00	7.85 ± 0.40	0.59 ± 0.02	0.32 ± 0.00	9 ± 0	0.51 ± 0.01	94 ± 2


*The thermokarst water sample was collected on 29 August 2010, and marine site water samples were collected on 26 April 2011 and 17 August 2011. A full survey of this system can be found in Baer et al. (2017). Numbers reported are the average of triplicate samples ± the standard deviation. BD = below detection or < 0.03 μmol N L^-*1*^. ND = not determined.*

Distinct chemical and biological differences in seawater were observed between April and August. Ambient Chl *a* and bacterial abundance concentrations were 3- to 4-fold higher in August than April (**Table 2**). Dissolved inorganic nutrient concentrations were higher in April than August, while DOM concentrations were lower (**Table 2**). The largest difference in ambient nutrient stocks occurred within the NO_3_^-^ pool, which was 26-fold higher in April than in August.

### Seasonal Differences in *In Situ* Bacterial Community Composition

**FIGURE 2:**
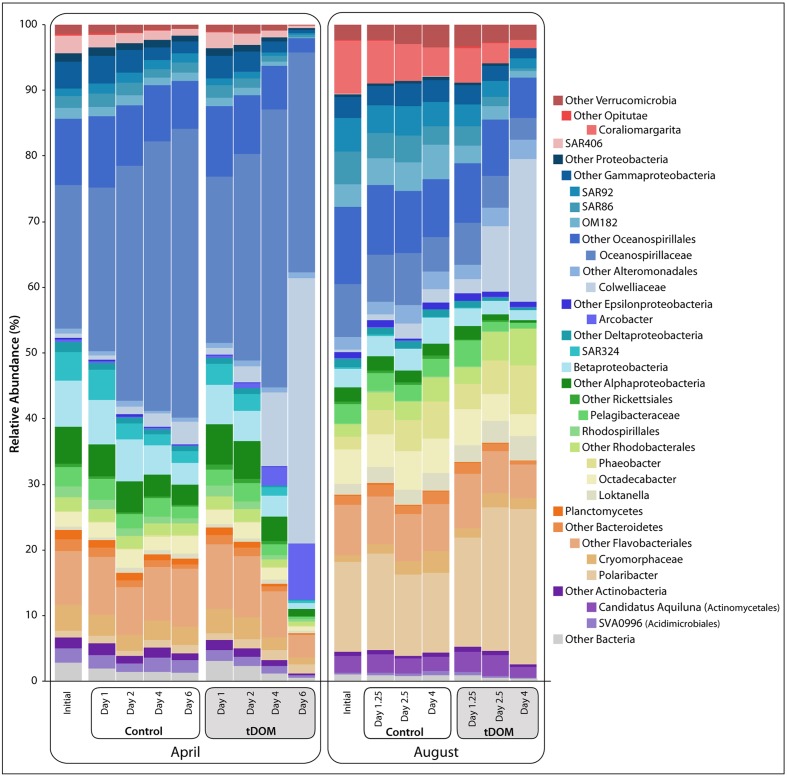
Bacterial relative abundance with tDOM additions. Stacked bar chart of bacterial taxonomy throughout the course of the bioassays [Controls and treatments with additions of terrestrial-derived dissolved organic matter (tDOM)]. Taxonomy was assigned in QIIME (v. 1.8) using the RDP classifier (Caporaso et al., 2010) based on DNA sequences, randomly subsampled to 3037 sequences per sample, from the v4 region of the 16S rRNA gene. Major bacterial phyla are further broken down into more specific taxonomic ranks (class or order) to highlight the dynamics of these taxa throughout the bioassays.

Distinct seasonal differences were observed across multiple taxonomic levels. The ambient communities used in the April and August bioassays shared fewer than 50% of their OTUs. The most abundant taxa classes were *Gammaproteobacteria*, *Betaproteobacteria*, *Alphaproteobacteria*, and *Deltaproteobacteria* within the phylum *Proteobacteria*, as well as phyla *Bacteroidetes, Verrucomicrobia*, and *Actinobacteria* (**Figure 2**). *Gammaproteobacteria* was the dominant group in both April and August, accounting for approximately 42% and 39% of the populations, respectively. OTUs related to family *Oceanospirillaceae* were the largest contributors to this class and constituted approximately 22 and 8% of the April and August communities, respectively. Sequences closely related to oligotrophic marine taxa (e.g., SAR86, SAR92 and OM182; Cho and Giovannoni, 2004) constituted only 4.5% of the April population but 13.5% of the starting populations in August. *Betaproteobacteria* were more prevalent in April (7%) than in August (∼3%), with dominant OTUs identified within the families *Comamonadaceae* and *Burkholderiaceae*. Although the total contribution of *Alphaproteobacteria* was similar between the two seasons (15.7 and 16.4% in April and August, respectively), contributions from unclassified *Alphaproteobacteria* OTUs were relatively more abundant in April and order *Rhodobacterales* OTUs had higher relative abundances in August. *Bacteroidetes*, dominated by *Polaribacter* spp., comprised only 1% of the April community compared to ∼13.7% in August. *Verrucomicrobia* (especially *Coraliomargarita* sp.) was also less abundant in April (<2%) than in August (∼10%).

Less abundant members of the starting bacterial communities, including the class *Deltaproteobacteria* (SAR324), and the phyla *Actinobacteria (Acidimicrobiales* sp., SVA0996 clade), and Marine Group A (SAR406), also exhibited distinct seasonal patterns. The proportion of SAR324, was 20-fold greater in April (4.3%) than in August (0.18%). Likewise, proportions of *Acidimicrobiales* were greater in April (2%) than in August (0.5%). Sequences closely related to SAR406 made up 2.7% of the April population but constituted a negligible proportion of the August community. In August, however, ∼2.5% of the *Actinobacteria* sequences were closely related to candidate genus “*Aquiluna*,” a genus that was rare in April.

### Biological and Chemical Changes in Bioassays

**FIGURE 3:**
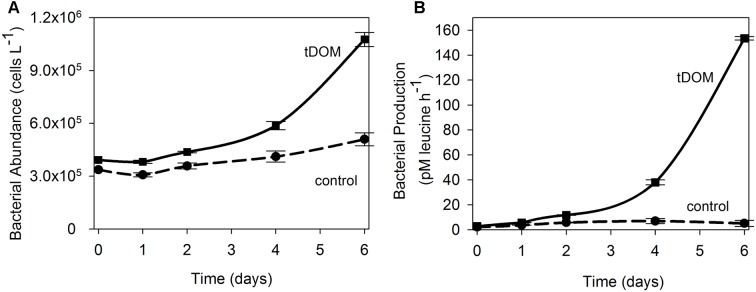
Bacterial response to tDOM additions. Time series of bacterial abundance **(A)** and bacterial production **(B)** data for the April bioassays where near coastal Arctic microbial communities were amended with terrestrially derived dissolved organic matter (tDOM). Data points are the mean (*n* = 2) ± half the range of duplicate samples. The tDOM treatment is depicted by the solid line and the Control treatment, which contains no additional DOM, is depicted by the dashed line.

**FIGURE 4:**
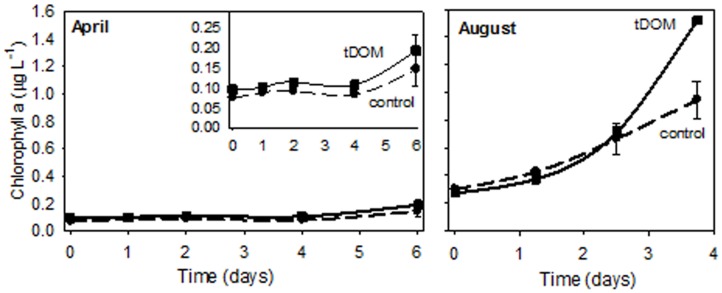
Chlorophyll a response to tDOM additions. Change in Chl *a* concentrations for the ice-covered (April) and open-water (August) bioassays. The inset in the April figure zooms in on the lower range of the Chl *a* scale. Control treatments are indicated by a dashed line and treatments with additions of tDOM are indicated using a solid line.

Additions of tDOM affected both ice-covered (April) and open-water (August) microbial communities. Bacterial abundance at the end of the April experiment was 3-fold higher when tDOM was added compared to the Control (**Figure 3A**). In April, bacterial production increased 2-fold in the Control and 55-fold in the tDOM treatment over the 6-day incubation (**Figure 3B**). Chl *a* concentration doubled in both the Control and tDOM treatments in April (**Figure 4**). In August, however, increases in Chl *a* concentration were nearly 2-fold higher (1.6 ± 0.0 μg Chl *a* L^-1^) in the tDOM treatment than in the no addition Control (0.9 ± 0.1 μg Chl *a* L^-1^; **Figure 4**).

**FIGURE 5:**
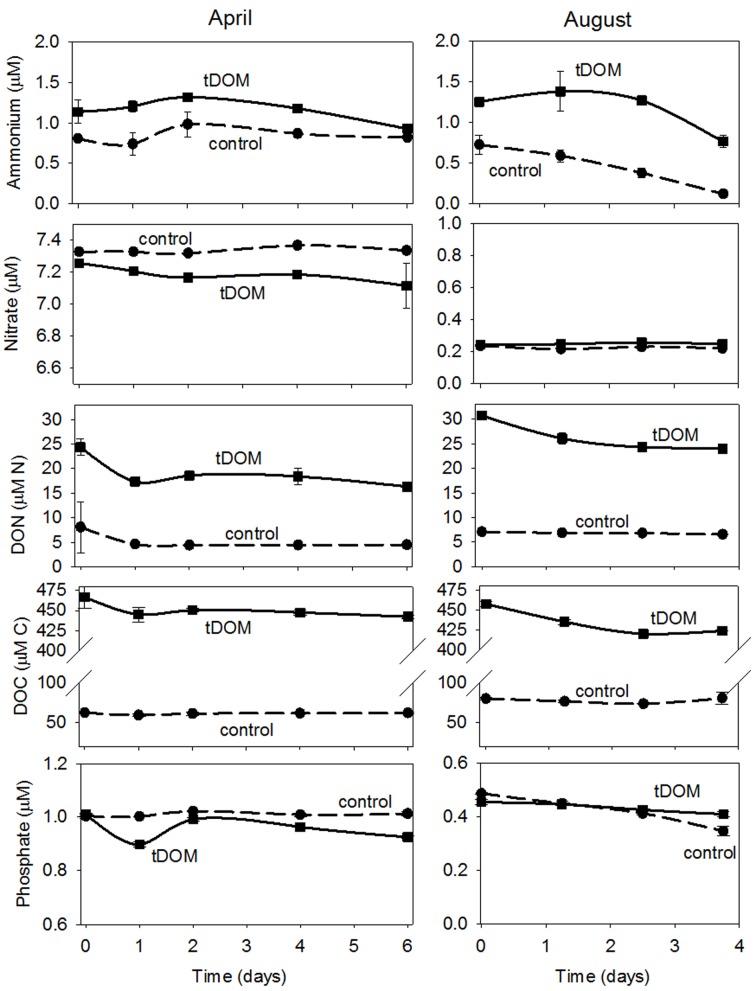
Bioassay nutrient concentrations. Time series nutrient concentration data for April and August bioassays where near coastal Arctic water was amended with terrestrially derived dissolved organic matter (tDOM). Data points are the mean (*n* = 2) ± half the range of duplicate samples. The tDOM treatment is depicted by the solid line and the Control treatment which contains no additional DOM, is depicted by the dashed line.

Little change in NH_4_^+^ concentration was observed in both the Control and tDOM treatments in April, but NH_4_^+^ concentrations decreased by approximately 0.5 μmol N L^-1^ in both the Control and tDOM treatments in August (**Figure 5**). Concentrations of NO_3_^-^ did not change in either treatment or season, even though NO_3_^-^ concentrations were an order of magnitude higher in April than August (**Figure 5**). DON concentrations in the tDOM treatments decreased by 7 ± 1 μmol N L^-1^ in April and by 8 ± 1 μmol N L^-1^ in August (**Figure 5**), compared to no change in the Control treatments. DOC concentrations in the tDOM treatments decreased by 29 ± 2 μM and 37 ± 3 μmol C L^-1^ in April and August, respectively (**Figure 5**), compared to no change in the Control treatments (**Figure 5**). Concentrations of PO_4_^3-^ decreased by 0.15 ± 0.04 μmol P L^-1^ in April and 0.3 ± 0.05 μmol P L^-1^ in August in the tDOM treatments, compared to no change in the Controls.

### Bacterial Community Response to tDOM Amendments

**FIGURE 6:**
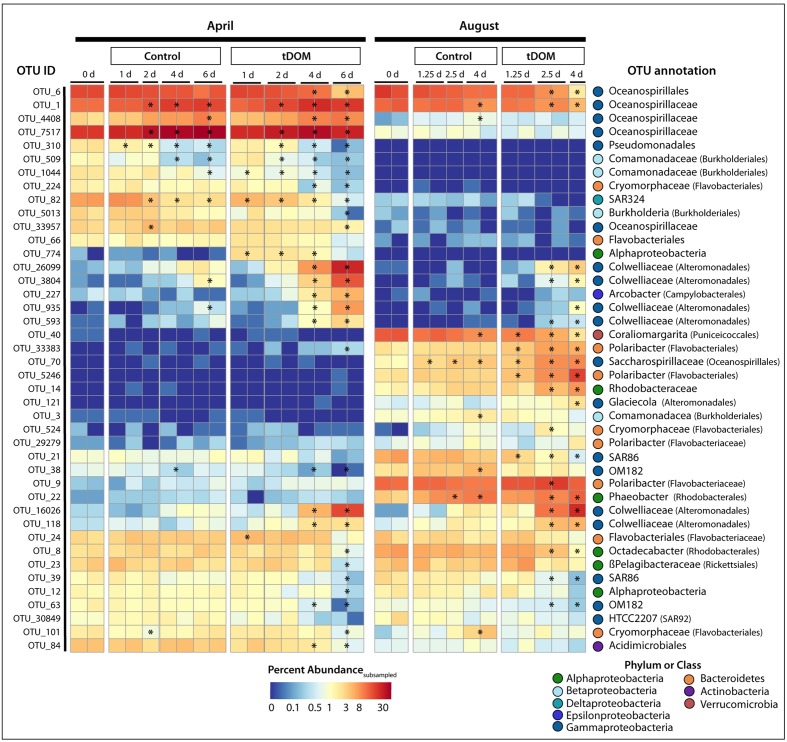
Change in operational taxonomic unit (OTU) relative abundance. Heatmap showing the percent abundance of OTUs identified to significantly change (adjust *p*-value < 0.05) in relative abundance over the course of the bioassays. Plotted are OTUs that were at least 1% of the total number of subsampled sequences in at least one sample. The OTU ID is noted to the left of the heatmap while the annotation, given as the lowest taxonomic level, is to the right of the heatmap. The bioassay (April or August), treatment (Control or tDOM) and timepoint (in days) are noted above the heatmap. Finally, for each OTU, asterisks within the heatmap denote a sample or pair of samples (duplicates) that have a relative abundance that is significantly greater or less than the Control.

A clear shift in community composition was observed in response to tDOM amendments (**Figure 2**), resulting in decreased bacterial diversity in all tDOM treatments (Supplementary Figure S1) and indicating that tDOM selected for and against certain species and clades. Bacterial diversity also decreased in control bioassays, but to a lesser degree, suggesting that bottle effects may also play a part in bringing about the observed declines in diversity. The tDOM bioassays diverged from the Control communities after 4 days in April and after 2.5 days in August (**Figure 2** and Supplementary Figure S2). A total of 138 bacterial OTUs (out of 1656) significantly (*p* < 0.05) increased or decreased during either April or August. Approximately 42 of these 138 OTUs (e.g., 30%) reached a relative abundance of > 1% in at least one sample, and belonged to phyla *Actinobacteria*, *Bacteroidetes*, *Proteobacteria*, or *Verrucomicrobia* (**Figure 6**). Sixteen of these 42 OTUs (38%) changed in either the Control or tDOM bioassays or both (**Figure 6**). The change in 14 of these 16 OTUs showed significant correlations (ρ > 0.5 or < –0.5 *n* = 16, *p* < 0.05) with either Chl *a* or inorganic nutrient concentrations indicating a response to phytoplankton growth or ambient nutrient stocks (Supplementary Figure S3). While 5 of these 16 OTUs were also correlated with DOC concentration, the correlations with Chl *a* were greater (ρ > 0.8 or < –0.7, *p* < 0.05). For 7 out of the 16 OTUs that changed in both Controls and treatments, the change in relative abundance in the tDOM bioassays was at least 2-fold higher in the tDOM treatments than in the Controls, indicating that at least some of the increase in relative abundance was due directly or indirectly to the tDOM addition.

**FIGURE 7:**
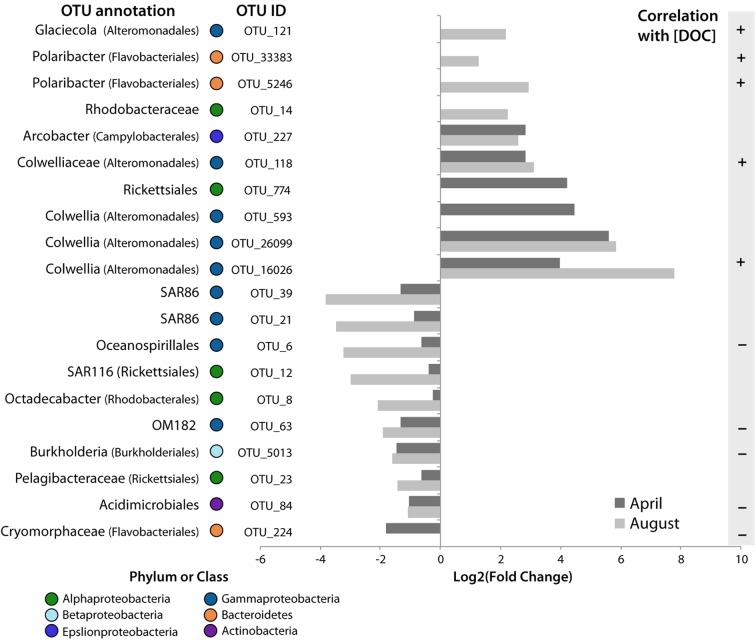
Potential sentinels of increasing tDOM. Log2 of the fold change in relative abundance of OTUs that were found to significantly increase or decrease in tDOM bioassays only. The amount of change at 4 days in each bioassay is plotted. Taxonomic classification of the OTU is noted to the right of the OTU# (more detailed classifications are provided in **Figure 2**). The presence of a significant (*p* < 0.05) correlation with DOC concentration is also indicated to the right of the plot. OTU_593 and OTU_33383 also increased in the April bioassay but this change could not be calculated since it was not present at the start of the experiment in these bioassays.

Here we highlight 20 OTUs that increased in the tDOM bioassays, but not in the Controls, and therefore, may be sentinels for increased terrestrial inputs to coastal Arctic systems (**Figure 7**). Specifically, 10 OTUs responded positively to the addition of tDOM and 5 of these were positively correlated with DOC concentrations (ρ > 0.56, *n* = 16 for each OTU, *p* < 0.05, **Figure 7**). The 10 OTUs that increased in relative abundance come from 2 bacterial phyla (**Figure 7**): *Bacteroidetes* (*Polaribacter* spp.) and three classes within the *Proteobacteria* (*Gammaproteobacteria*, *Alphaproteobacteria*, and *Epsilonproteobacteria*).

Of the *Gammaproteobacteria* that significantly increased in the presence of tDOM, four OTUs, which increased in both April and August, were closely related to the family *Colwelliaceae* [OTU_118, OTU_593, OTU_26099, OTU_16026; (**Figure 6**)]. Three of these OTUs were closely related to the genus *Colwellia* (**Figure 7**). Another gammaproteobacterial taxa that increased in response to tDOM, but only in August, was an OTU related to *Glaciecola sp.* (OTU_121). While we observed a decline in some groups of marine Roseobacter clade (e.g., *Octadecabacter* sp.; OTU_8) in the tDOM bioassays, discussed in greater detail below, we observed an increase in other taxa, especially one related to order *Rhodobacterales* (OTU_14). The relative abundance of an OTU closely related to *Arcobacter*, an *Epsilonproteobacteria*, increased in response to tDOM in both seasons (**Figure 7**).

While the aforementioned OTUs benefited from tDOM, the relative abundance of 10 other OTUs decreased in the tDOM treatment, but not in the Controls. The relative abundance of half of these OTUs were inversely correlated with DOC concentrations (ρ < –0.54, *n* = 16, *p* < 0.05), suggesting increased DOM inputs impede the growth of these OTUs relative to other members of the community. The negatively affected OTUs included taxa within *Gammaproteobacteria*, *Alphaproteobacteria*, *Bacteroidetes*, *Betaproteobacteria*, and *Actinobacteria*.

Several marine clades of *Gammaproteobacteria*, including SAR86 (OTU_39, OTU_21), OM182 (OTU_63) and an *Oceanospirillales*-related OTU (OTU_6) declined significantly (*p* < 0.05) with the addition of tDOM. Three *Alphaproteobacteria* OTUs (OTUs 8, 12, and 23) showed a significant decline in relative abundance in the *tDOM*-amended bioassays, including an OTU belonging to the family *Pelagibacteraceae* (OTU_23), an *Octadecabacter* sp. (OTU_8), and a member of the SAR116 clade (OTU_12). Within the *Bacteroidetes* phylum, an OTU closely related to family *Cryomorphaceae* (OTU_224, with 100% identity to the Antarctic fosmid ANT39E11; Grzymski et al., 2006) was found to decline throughout the course of the April bioassay.

## Discussion

Rivers, estuaries and coastal regions are particularly important for the transformation and degradation of tDOM (Raymond and Bauer, 2000; Battin et al., 2008). Approximately half of the DOC released by Arctic rivers is removed over the continental shelves (Letscher et al., 2011). This project explored the effects of increased tDOM on coastal Arctic microorganisms under ice-covered and ice-free conditions. By investigating the effect of tDOM on microbial community composition we found that additions of tDOM selected for certain species and against others, altering community composition and decreasing bacterial diversity. Increases in tDOM discharge to the coastal Arctic will change both the biogeochemistry and microbial composition of coastal regions.

### Bulk Microbial Response to and Lability of tDOM

Concentrations of Chl *a* increased in all treatments indicating that the autotrophic communities were not nutrient limited at the time of collection. The lower initial Chl *a* concentration and low light level (4.7 μmol quanta^-1^ m^-2^ s^-1^) may have contributed to the lack of an additional response to the tDOM supplement in April. The divergence between the Chl *a* concentrations in the Control and in the tDOM treatments in August occurred after incubating for 3 days. Although phytoplankton may have been capable of directly using tDOM, the 3 day delay in growth may indicate that regenerated nutrients or critical microbial byproducts contributed to the observed change in Chl *a*. Tank et al. (2012) estimated that remineralized riverine N may support up 0.5 to 1.5 Tmol C yr^-1^ of primary production.

To further support the importance of remineralized nutrients in Arctic microbial growth, we found that reduced forms of N (i.e., NH_4_^+^ and DON) were more labile to the receiving community than NO_3_^-^ which did not change in concentration in either treatment or season on the timescale tested, even though it was an order of magnitude higher in ambient waters in April than in August (**Figure 5**). This hypothesis is supported by high regeneration rates (15.2 nmol N L^-1^ h^-1^) and NH_4_^+^ uptake rates (14.1 nmol N L^-1^ h^-1^) observed in a parallel study for this specific microbial community (Baer et al., 2017). Concentrations of NH_4_^+^ decreased by approximately the same concentration (0.5 μM N) in both the Control and tDOM treatments in August, however, DON may have been remineralized into NH_4_^+^ and then quickly used by the community with no net change in concentration. Changes in nutrient concentrations are often not observed in tightly coupled regenerative systems (Sipler and Bronk, 2015).

Approximately 7 – 8% of the DOC supplied in this study was removed during the incubations. Because no change in the bulk DOC concentration was observed in the control treatments from either season it is likely that the majority of DOC lost during the incubation in the tDOM treatments was from the tDOM pool. The lability of the tDOM source to the summer community is possibly underestimated as phytoplankton growth in the tDOM treatment was observed for the final time point and plankton derived DOC production may have masked additional tDOM removal. The timing restrictions of our field season limited the duration of our incubations but it is likely, based on changes in bacterial abundance in spring and increasing Chl *a* concentrations at the final time point in summer, that additional degradation would occur with longer incubation times.

The 7 – 8% lability presented here is lower than riverine tDOM lability reported by other incubation studies (17 – 62%; Holmes et al., 2008; Mann et al., 2012; Vonk et al., 2013; Spencer et al., 2015). Some of this difference may be due to seasonal or regional differences in composition. For example, a study focusing on the lability DOC from Alaskan Arctic rivers showed DOC collected between July and August was ∼2 –9% labile when incubated in the dark for 90 days (Holmes et al., 2008). This proportion is similar to the 7 – 8% that we observed. Another study conducted in the Russian Arctic found that >50% of DOC in permafrost thaw streams collected in September was bioavailable to riverine bacterial communities on short 7 day time scales (Spencer et al., 2015). The distinct difference in lability over similar time scales between our study and Spencer et al. (2015) could be due to differences in the target microbial communities or DOM composition. For example, our study investigated the response of coastal marine communities while the other aforementioned studies focused on to freshwater riverine communities. The high bacterial production rates show that the community in our study was primed to use DOM but based on the complex compositional characteristics of the tDOM source many of the compounds supplied were likely less easily degraded than simple amino acids used for production estimates. Therefore it is expected that the rate of degradation decreased as the most labile fractions are removed (Cherrier et al., 1999).

Unfortunately, data showing changes in DOM composition over the incubation period for our study are not available and direct comparisons of the FT-ICR MS data between our study and Spencer et al. (2015) are difficult as samples were analyzed in different ionization modes. What we can determine from comparing these studies is that a large proportion of compounds in both sources showed elemental characteristics of being moderately unsaturated and lignin-like, and both showed district clustering of some of the compounds in the lipid/ protein-like or aliphatic region of the van Krevelen diagram (**Figure 1**). What cannot be determined from our current data is how many of these compounds are shared among both sites/ regions and if the same compounds are labile to both communities.

Another difference between our study and the previous studies is that we did not exclude phytoplankton from our incubations and incubated in the presence of light. The inclusion of primary production could mask the reduction of riverine DOC by supplying an additional source of DOC to a closed system but their presence also provides a more inclusive microbial response to tDOM additions by including both autotrophic and heterotrophic processes. We acknowledge that, like most bioassay studies, our conclusions are limited by the exclusion of higher trophic levels but feel that important information can be gained about potential impacts on the base of the coastal food web from our work assessing the response of the phytoplankton and bacterial community.

### Seasonal Differences in *In Situ* Microbial Community Composition

The extreme physical and chemical differences observed between April and August likely contributed to the distinct variations between the April and August ambient microbial communities. Large seasonal changes in organic matter composition are observed throughout the Arctic (e.g., Crump et al., 2003; Connelly et al., 2015; Mann et al., 2016), and can influence the composition (Crump et al., 2003; Judd et al., 2006) and physiology of the ambient microbial community (Sipler et al., 2017). An evaluation of offshore sites found differences in community composition between seasons to be insignificant (Kirchman et al., 2010), which when combined with our results, further supports the importance of terrestrial influence (tDOM) in shaping coastal microbial communities. The observed variations in community composition are likely influenced by the dramatic seasonal changes in their physiochemical environment. Given the notable differences in the ambient microbial community compositions in April and August, it follows that the microbial response to tDOM will differ seasonally as well.

*Gammaproteobacteria* were the dominant phylum in both April and August. The largest contributors in April were OTUs related to the *Oceanospirillaceae* family. Members of this family have been shown to support phytoplankton growth in polar waters through the synthesis of cobalamin (vitamin B12) (Bertrand et al., 2015). Sequences closely related to open-ocean marine taxa (e.g., SAR86 and OM182 (Cho and Giovannoni, 2004) were more prevalent in the nutrient-poor August waters, similar to observations observed by Ghiglione et al. (2012) in Arctic and Antarctic waters.

*Betaproteobacteria* were more prevalent in April than in August. Members of *Betaproteobacteria* have been observed throughout Arctic surface waters, especially in the summer (Garneau et al., 2006; Ghiglione et al., 2012) and are often indicators of freshwater inputs. Therefore, their higher relative abundance in April, before the spring freshet, was unexpected. However, a recent study in the Beaufort Sea found that *Betaproteobacteria* composed up to 20% of bacterial cells sampled from ice-covered waters in February (Alonso-Sáez et al., 2014), suggesting the niche for different members of *Betaproteobacteria* is wider and potentially more diverse than previously thought.

Proportions of *Alphaproteobacteria* were similar between the two seasons. *Phaeobacter* sp. (*Roseobacter* clade), which was more dominant in August, has been shown to grow in association with phytoplankton (Brinkhoff et al., 2008). They promote algal growth through the synthesis and secretion of antibiotics and algal growth stimulants (Seyedsayamdost et al., 2011).

*Bacteroidetes*, dominated by *Polaribacter* spp., comprised ∼13.7% of the August community. *Polaribacter* are abundant and active members of coastal Arctic and Antarctic bacterial communities (Malmstrom et al., 2007; Ghiglione et al., 2012; Grzymski et al., 2012; Nikrad et al., 2012). Summer conditions appear to favor taxa belonging to the Polaribacter genus. For example, the proportion *Polaribacter* active in the uptake of simple DOC substrates is 7- to 10-fold higher in summer compared to winter (Nikrad et al., 2012). Several *Polaribacter* taxa have been observed to have gas vesicles (Gosink et al., 1998) and contain proteorhodopsin (González et al., 2008; Grzymski et al., 2012). Given their high relative abundances and notable activity in polar summer waters as well as their buoyancy-related adaptations, members of this taxa likely play a key role in summer C cycling in coastal Arctic ecosystems, perhaps benefiting from long days.

*Verrucomicrobia* (especially *Coraliomargarita* sp.) was another phylum that dominated August samples. The majority of *Verrucomicrobia* sequences in our samples belong to subdivision 4, which are observed in surface waters globally (Freitas et al., 2012). *Verrucomicrobia* have been shown to increase in relative abundance in response to phytoplankton-derived DOM inputs (Landa et al., 2014) and may be important polysaccharide degraders in marine environments (Martinez-Garcia et al., 2012).

Although constituting a smaller proportion of the starting communities, *Deltaproteobacteria* (SAR324), *Actinobacteria (Acidimicrobiales* sp., SVA0996 clade), and SAR406 also exhibited distinct seasonal patterns. The proportions of two taxa, SAR324 and *Acidimicrobiales*, that are important in winter N cycling in these waters (Connelly et al., 2014), were greater in April than in August. The higher abundance of SAR324 in April is not surprising as it is known to be a metabolically flexible taxa able to take advantage of diverse nutrient sources (Sheik et al., 2014). SAR406 was more abundant in April but negligible in August. Sequences of SAR406 have been recovered under the sea-ice east of our Barrow field site in the Beaufort Sea (Collins et al., 2010) and in surface waters elsewhere in the Arctic and Antarctic (Ghiglione et al., 2012). Both SAR324 and SAR406 tend to be present in higher abundances in deep waters, yet have also been observed in winter polar surface waters (Ghiglione et al., 2012 and this study), suggesting that their distribution is not limited to deep waters, especially in polar environments. A metagenomic assessment of Antarctic surface waters also highlighted the dominance of chemolithoautotrophic pathways characteristic of these taxa in winter surface waters (Grzymski et al., 2012). The actinobacterial sequences detected in August were closely related to candidate genus “Aquiluna,” a genus that was rare in April. Originally thought to be a freshwater genus (Hahn, 2009), sequences closely related to “ca. Aquiluna” have been recovered from marine environments, including from the Arctic (Kirchman et al., 2010; Kang et al., 2012) and have been observed to contain actinorhodopsin, suggesting the potential for photoheterotrophy by this taxa (Kang et al., 2012).

### Bacterial Response to tDOM Amendments

Additions of tDOM selected for certain bacterial taxa and against others, altering community composition and decreasing bacterial diversity. Members of *Actinobacteria*, *Bacteroidetes*, *Proteobacteria*, and *Verrucomicrobia* significantly responded to the addition of tDOM. Twenty OTUs were identified as potential sentinels for increased terrestrial inputs in coastal Arctic systems (**Figure 7**). *Polaribacter* spp. within *Bacteroidetes*, taxa within *Gammaproteobacteria* (*Colwelliaceae* family and *Glaciecola* spp.), a member of the alphaproteobacterial family Rhodobacteraceae, and an *Epsilonproteobacteria* related to *Arcobacter* sp. all increased in relative abundance in the presence of tDOM (**Figure 7**). Many of the taxa that responded positively to the tDOM additions are common within polar waters, are known to grow in association with phytoplankton, and thrive in high nutrient environments. Common in polar waters in both spring (Malmstrom et al., 2007) and summer (Ghiglione et al., 2012; Grzymski et al., 2012), *Polaribacter* have been linked to the uptake and decomposition of complex polymeric DOM (Kirchman, 2002). Members of this clade are also often observed in association with polar phytoplankton blooms (Teeling et al., 2012; Landa et al., 2016). Here we extend evidence of their success to high-tDOM environments. Given their ability to thrive in the presence of phytoplankton-derived DOM *or* tDOM, the *Polaribacter* genus may be resilient to the ongoing changes in coastal Arctic waters.

The *Gammaproteobacteria* that significantly increased in the presence of tDOM were closely related to the family *Colwelliaceae* (**Figure 6**), specifically the genus *Colwellia* (**Figure 7**). Although this family was not very abundant in the starting communities in this study, it is clear that they may become dominant members of polar microbial communities during times of high DOM inputs. *Colwellia* has a diverse array of metabolic strategies aiding its survival in Arctic waters including blooming in response to phytoplankton-derived DOM (Landa et al., 2016), assimilating bicarbonate (Alonso-Sáez et al., 2010), production of extracellular enzymes capable of degrading high molecular weight compounds (Methé et al., 2005), degrading sinking organic matter (Delmont et al., 2014), and responding to tDOM inputs as described in this study. *Glaciecola* sp. (*Gammaproteobacteria*) also increased in response to tDOM. This genus is capable of growing in association with complex DOM sources. For example, this genus has been found in sea ice (Brakstad et al., 2008) and sea water contaminated with crude oil (Chronopoulou et al., 2015), appearing to be able to survive in systems with high hydrocarbon concentrations. Arctic peats naturally contain hydrocarbons (Yunker et al., 1993); therefore, it is not surprising that 16% of the DOM compounds within the tDOM source had elemental characteristics similar to Aromatics and 15% as condensed Aromatics that could have provided a source of DOM for *Glaciecola* sp.

Roseobacters are a metabolically diverse clade of *Alphaproteobacteria* (Buchan et al., 2005; Newton et al., 2010). While we observed a decline in some groups of Roseobacter (e.g., *Octadecabacter* sp.) in the tDOM bioassays, discussed in greater detail below, we observed an increase in other taxa, especially an OTUs related to *Rhodobacterales*, further highlighting the metabolic breadth of this important clade of marine bacteria. Our results suggest that some taxa within the globally important *Roseobacter* clade will thrive in a changing Arctic Ocean.

*Arcobacter*, an *Epsilonproteobacteria*, also increased in response to tDOM. In marine environments, *Arcobacter*-related sequences are often associated with metazoans (Gugliandolo et al., 2008; Fontanez et al., 2015), yet, the *Arcobacter* that responded to tDOM was more closely related (96% similar) to a sulfur-oxidizer (*Candidatus* Arcobacter sulfidicus) (Wirsen et al., 2002). Because waters between 1 and 18 m depth account for 17% of Arctic shelves (including the site where our seawater samples were collected), and because wave action is expected to increase in the coastal Arctic due to sea ice loss (Overeem et al., 2011), taxa that thrive on tDOM or near sediments (e.g., sulfur-oxidizers) may become more dominant in the water column in the future.

While several OTUs benefited from tDOM, others, including taxa within *Gammaproteobacteria*, *Alphaproteobacteria*, *Bacteroidetes*, *Betaproteobacteria*, and *Actinobacteria*, were negatively affected by the presence of tDOM. More than half of the OTUs that decreased in relative abundance in the tDOM bioassays were inversely correlated with DOC concentrations. This suggests that increased DOM inputs will impede the growth of these OTUs relative to other members of the community.

Several marine clades of *Gammaproteobacteria* that declined with the addition of tDOM have previously shown diverse responses to nutrients and DOM sources. For example, SAR86, which has been shown to have mixed responses to phytoplankton blooms (Obernosterer et al., 2011; Tada et al., 2011; Rinta-Kanto et al., 2012; Wemheuer et al., 2014; Landa et al., 2016), are found in higher concentrations in the open ocean rather than in Arctic coastal waters (Ghiglione et al., 2012). Likewise, members of the OM182 clade exhibit higher growth rates in lower C environments (Cho and Giovannoni, 2004). Given their preference for oligotrophic waters, it is not surprising that SAR86 and OM182 OTUs decreased in the tDOM bioassays. Similar to SAR86, members of the *Oceanospirillales* order have shown mixed responses to DOM amendments suggesting global variation in how these taxa, or ecotypes within these taxa, respond to tDOM inputs. Some studies observed a response of *Oceanospirillales* to both phytoplankton-derived and plant-derived organic matter (Mou et al., 2008), but other studies show that some members of this order decrease in relative abundance in response DOM amendments (Landa et al., 2013). The *Oceanospirillales*-related OTU in our study was negatively correlated with DOC concentration and significantly decreased in both the April and August tDOM incubations.

This study highlights the ecological complexity of Arctic microbes. As discussed above, some OTUs that are known to respond positively to phytoplankton blooms and presumably phytoplankton derived DOM decreased in relative abundance while others increased. Having access to preferred forms of DOM does not mean that the other biogeochemical conditions remain favorable enough for certain species to take advantage of the resource. High levels of DOM, and the metals that they chelate may (1) inhibit some species that benefit from the lower concentration tDOM additions or (2) species that benefit from tDOM additions may simply outcompete or actively inhibit other species neutral to tDOM additions. The underlying mechanisms that control microbial community composition are not fully understood but these mechanisms are likely much more complex than C availability alone.

*Alphaproteobacteria*, including OTUs belonging to the *Pelagibacteraceae* family, an *Octadecabater* sp., and a member of the SAR116 clade, also showed a significant decline in relative abundance in the tDOM amended bioassays. *Pelagibacteraceae* taxa specialize in the degradation of fresh, low molecular weight dissolved organic matter (Malmstrom et al., 2005; Poretsky et al., 2010), while the tDOM in this study represents a complex mixture of predominantly lignin -like compounds (**Table 1**), that may not favor the growth of *Pelagibacteraceae* or other oligotrophs. Similarly, *Octadebacter* sp. appear to lack the machinery needed to degrade aromatics (Newton et al., 2010), an important characteristic for utilizing many of the tDOM compounds. Paralleling our observations, SAR116 activity was observed to be low in eutrophic temperate coastal waters (Campbell et al., 2009) and ribotypes of this clade have been observed to have higher abundances in open ocean waters during highly oligotrophic periods (Morris et al., 2005).

Within the *Bacteroidetes* phylum, an OTU closely related to the *Cryomorphaceae* family (with 100% identity to the Antarctic fosmid ANT39E11; (Grzymski et al., 2006) was found to decline throughout the course of the ice-covered bioassay despite members of this family being known to thrive in high organic matter environments. Members of this taxa, as well as SAR86, the order *Oceanospirillales* and the *Pelagibacteraceae* family, have been previously shown to thrive in high DOM environments but the observed decrease in our samples highlights the importance of not generalizing an organism’s preference for high organic matter environments to all types or sources of organic matter. Even microorganisms that thrive in high DOM waters may not do well in polar waters with a high fraction of terrestrial or tundra-derived organic matter.

Although we have focused on OTUs that only responded positively or negatively to the tDOM additions, approximately half of the OTUs that changed in both treatments, increased more in the tDOM treatments than the Controls, indicating that at least a fraction of their increase in relative abundance was due directly or indirectly to the tDOM addition and not solely attributed to bottle effects. For example, commonly considered generalist taxa (Mou et al., 2008), OTUs belonging to the gammaproteobacteria family *Colwelliaceae* increased in both Control and tDOM bioassays in our study, but the increase in the tDOM bioassays far exceeded that in the Control bioassays (**Figure 6**), highlighting their potential importance in taking advantage of increasing inputs of tDOM in a changing Arctic Ocean. Because phytoplankton (Chl *a*) increased in the August tDOM treatments it is hard to differentiate the direct impacts of tDOM or the indirect influence of tDOM promoting phytoplankton growth/ exudates. We argue that this study addresses the overall response, direct and indirect, of target coastal microbial communities to tDOM additions. Several of the OTUs that changed in both the Control and tDOM bioassays significantly correlated with Chl *a* concentrations or inorganic nutrient concentrations validating their response to phytoplankton growth or ambient nutrient stocks (Supplementary Figure S3). Similarly, while alpha diversity declined in both the Control and tDOM treatments, the magnitude of these decreases was greater in tDOM treatments. This suggests that tDOM amendments enhanced selection for bacterial taxa beyond that which was observed in the controls alone indicating that this selection was not only the result of bottle effects.

Seasonal differences in how quickly microbial communities responded to tDOM were apparent, with August tDOM-amended community composition diverging from control communities more rapidly than April communities (Supplementary Figure S2). It is possible that some of these differences may be temperature driven, given that small increases in temperature can have notable effects microbial metabolism (e.g., Kirchman et al., 2005), but our experimental design does not allow for this effect to be quantified. Nevertheless, our results suggest that summertime microbial communities may be poised to respond more rapidly to pulses of tDOM than communities in early spring. Regardless of season, the addition of tDOM generally shifted bacterial communities toward more copiotrophic and away from more oligotrophic taxa. No one order was found to respond universally (positive or negative) to the tDOM addition. These data suggest that not all sources of DOM (phytoplankton DOM vs. tDOM) are created equal and generalization about the potential impact of a source (e.g., DOM) to a specific phylum, family or clade could be misleading.

### Implications for a Warmer More tDOM Rich Arctic

We know that Arctic atmospheric temperatures are rising (Serreze and Francis, 2006; Screen and Simmonds, 2010), that permafrost reserves are thawing (Smith et al., 2005, 2010), and that tDOM inputs to the coastal Arctic are increasing (Fichot et al., 2013). Here we provide evidence that these increases in tDOM to coastal waters of the Arctic Ocean will likely yield a shift in microbial community composition. What remains unknown is how all of these changes will impact global C, N, and phosphorus (P) cycles.

The most obvious impact for the global C cycle is the release of carbon dioxide associated with the respiration and degradation of this abundant and increasing source of organic C. However, a less obvious consequence for the C cycle includes a potential changes in autotrophic and heterotrophic C-fixation. Several studies suggest that increased freshwater discharge will cause an overall reduction in coastal production (e.g., Balch et al., 2012, 2016; Wikner and Andersson, 2012); however, the present experiment provides evidence that some late season Arctic phytoplankton may benefit from tDOM additions through regenerated nutrients. No such benefit was observed in spring when light levels are low. Therefore, the positive Chl *a* response observed at the final summer time point may be indicative of a seasonal or community dependent response but that the overall impact on production may be negative.

The light absorption by the tDOM ranges into the visible spectrum, which decreases light needed for photosynthesis (Arrigo and Brown, 1996). The light attenuation associated with tDOM may be more physiologically consequential in spring when light is already limiting productivity (Sipler et al., 2017). A companion study Sipler et al. (2017) found that the addition of tDOM reduced C-fixation during a 24 h incubation. Additionally, several of the above-described *Alpha*- and *Gammaproteobacterial* taxa, including *Octadecabacter* and *Oceanospirillales* spp., that declined in abundance when tDOM was added, are important heterotrophic dissolved inorganic C-fixers (DeLorenzo et al., 2012). While other heterotrophic C-fixers, such as *Colwellia* sp. (Alonso-Sáez et al., 2012) or *Polaribacter sp.* (DeLorenzo et al., 2012), were observed to increase in response to tDOM, indicating increasing tDOM inputs may have mixed effects on heterotrophic C-fixation. Although autotrophic C-fixation dominates Arctic primary productivity, heterotrophic C-fixation may be important in seasons or regions (depth) when light is limiting (Alonso-Sáez et al., 2010; Herndl and Reinthaler, 2013).

Indirect impacts of DOM on primary production may also include promoting (Bertrand et al., 2015; Mishamandani et al., 2016) or potentially inhibiting co-existent or mutualistic bacterial species essential for phytoplankton growth. For example, reductions in specific taxonomic groups (e.g., *Oceanospirillaceae* sp.) may lead to reductions in bacterially synthesized compounds, including cobalamin, that are essential for phytoplankton growth (Bertrand et al., 2015). Thus, a decline in the abundance and activity of these organisms in an Arctic Ocean faced with increased inputs of tDOM may impact not only the cycling of organic C but also the sequestration of inorganic C.

Nutrient budgets will also be affected by increases in tDOM. A recent study investigated the potential for regenerated terrestrial-dissolved organic nutrients to balance Arctic Ocean inorganic nutrient budgets (Torres-Valdés et al., 2016). Although DON inputs appear insufficient in supplying enough N to offset the rather large denitrification deficit (Chang and Devol, 2009) in the Arctic Ocean, terrestrial DON and DOP sources do play an important role in Arctic and North Atlantic N and P cycles. Because microbial communities mediate the magnitude and rate of DOM regeneration, understanding how DOM and microbial pools will change in relationship to one another is critical to predicting the impacts of future environmental perturbations and trends (Kujawinski et al., 2016).

The extent and magnitude at which tDOM inputs increase in the Arctic may determine the future productivity of this sensitive ecosystem. In this interconnected system (Wassmann et al., 2011), changes in phytoplankton and bacterial community composition have enormous consequences for higher trophic levels locally and across the Arctic. Beyond the community changes we observed in response to tDOM amendments, more work is necessary to elucidate how these changes affect community metabolism, ecosystem function, and process rates important in the cycling of globally important elements.

## Conclusion

Changes in heterotrophic production and microbial community composition are expected as substantial reserves of tDOM, released from once stable permafrost, become available to coastal microbial communities. Unlike sea ice melt, which has been speculated to cause short term increases in primary production, a large scale loading of a C-rich source may increase carbon dioxide release via respiration and impact the cycling of both organic and inorganic nutrients. Here we have shown that the addition of tDOM promoted certain species and inhibited others, decreased bacterial diversity, thus shifting the microbial community composition. At least 7% of the terrestrial-DOC supplied during this study was consumed by both ice-covered and ice-free microbial communities. The terrestrial-DON fraction was proportionately more labile with ∼ 38% of the supplied DON being removed during the relatively short (4 – 6 day) incubations. Through this effort we have identified 20 OTUs that significantly changed in response to the tDOM addition that may serve as sentinels for future environmental change. Our work is a step forward in identifying microbial shifts that may indicate larger future impacts. Additional studies are needed to investigate temporal and spatial trends of these species as they relate to coastal humification, to confirm their role as true sentinels of change in the Arctic.

## Author Contributions

RS, DB, and TC designed the study; RS, DB, TC, and QR collected the samples; RS, CK, TC, and QR analyzed the data; RS, CK, and TC wrote the paper; all authors helped interpret the data and edited the paper.

## Conflict of Interest Statement

The authors declare that the research was conducted in the absence of any commercial or financial relationships that could be construed as a potential conflict of interest.

## Footnotes

1 http://www.earthmicrobiome.org/emp-standard-protocols/

2 http://www.genomesonline.org/

3 http://greengenes.secondgenome.com/

4 http://www.umass.edu/landeco/teaching/ecodata/labs/biostats.pdf
