# Supplementary material for: Microbial Community Response to Terrestrially Derived Dissolved Organic Matter in the Coastal Arctic
Source: Front Microbiol. 2017 Jun 9;8:1018. doi: 10.3389/fmicb.2017.01018 (PMC5465303; doi:10.3389/fmicb.2017.01018)
Supplement: Supplementary file 1 [file Data_Sheet_1.PDF]

Supplementary Material

Microbial Community Response to Terrestrially-derived Dissolved Organic Matter in the Coastal Arctic

Rachel E. Sipler [1*], Colleen T. E. Kellogg[2], Tara L. Connelly[3,†], Quinn N. Roberts[1], Patricia L. Yager[3], and Deborah A. Bronk[1]

[1]The Virginia Institute of Marine Science, College of William & Mary, Gloucester Point, VA, USA,

[2] Department of Microbiology & Immunology, University of British Columbia, Vancouver, BC, Canada,

[3] Department of Marine Sciences, University of Georgia, Athens, GA, USA

*** Correspondence:** Rachel Sipler: rachelsipler@gmail.com

Supplementary Figures

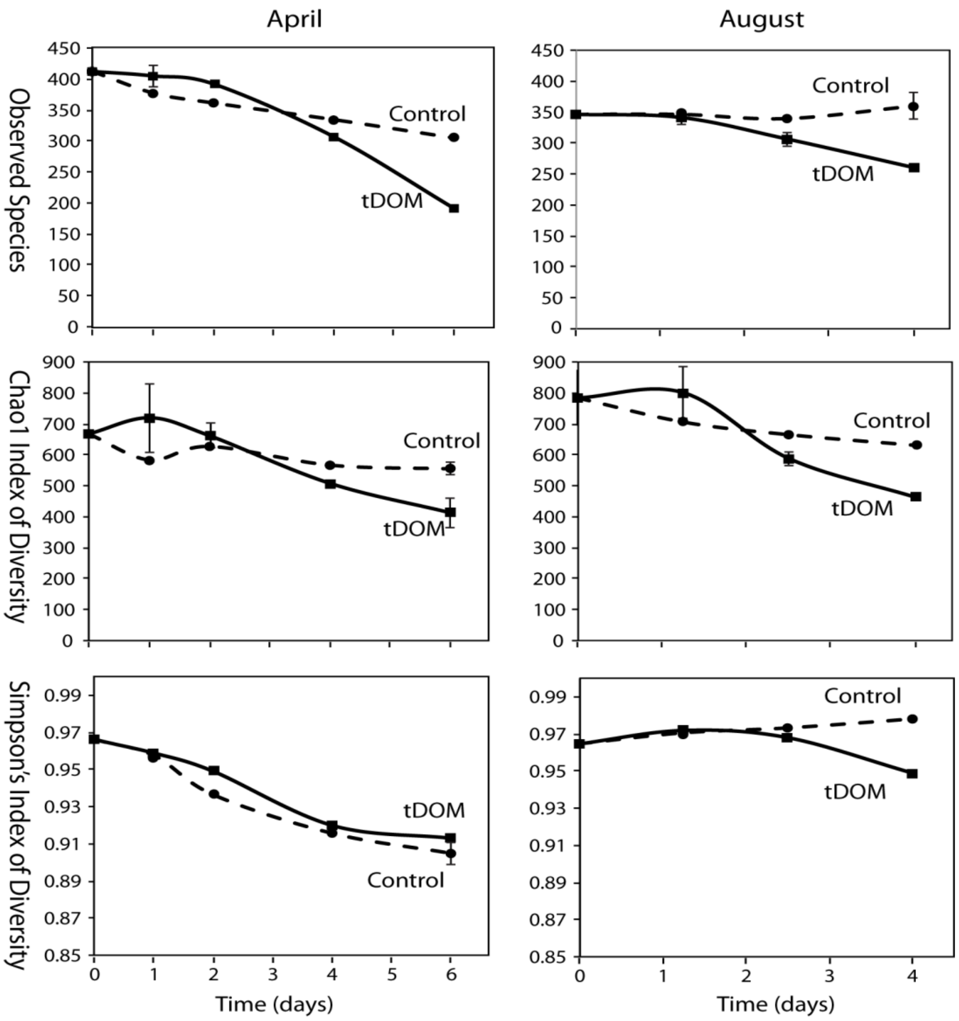

FIGURE S1 | Impact of tDOM on microbial diversity. Time series of alpha diversity for April and August bioassays where near coastal Arctic water was amended with terrestrially derived dissolved organic matter (tDOM). Data points are the mean (n=2) ± half the range of duplicate samples. The tDOM treatment is depicted by the solid line and the Control treatment which contains no additional DOM, is depicted by the dashed line. Three measures of alpha diversity were used (Observed Species, Chao1 index of diversity and Simpson's Index of Diversity) and were calculated in QIIME using the subsampled OTU table.

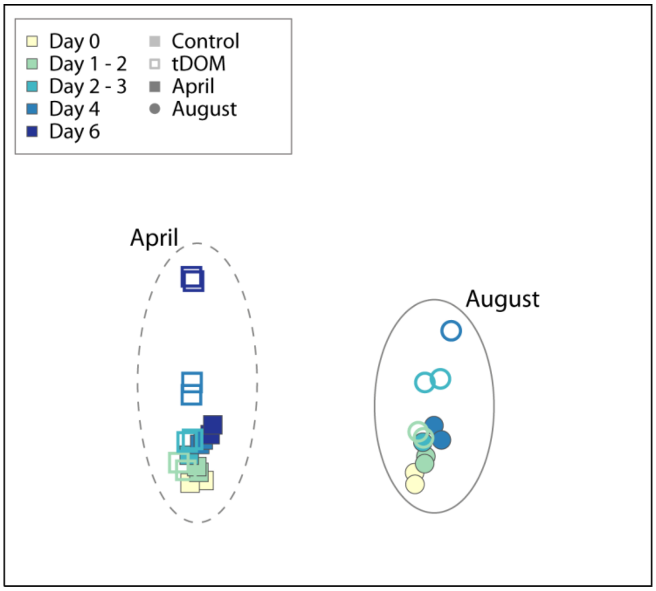

FIGURE S2 | Impact of tDOM on microbial diversity. Nonmetric multidimensional scaling (MDS) plot of the Bray-Curtis dissimilarity comparing subsampled bacterial community composition among treatments and bioassays. Abundances were transformed using the Hellinger transformation prior to calculating the Bray-Curtis dissimilarity. Filled symbols represent communities from the control treatments while open symbols represent communities from the tDOM treatments. Symbol shape indicates the month during which the bioassay was run (squares = April and circles= August).

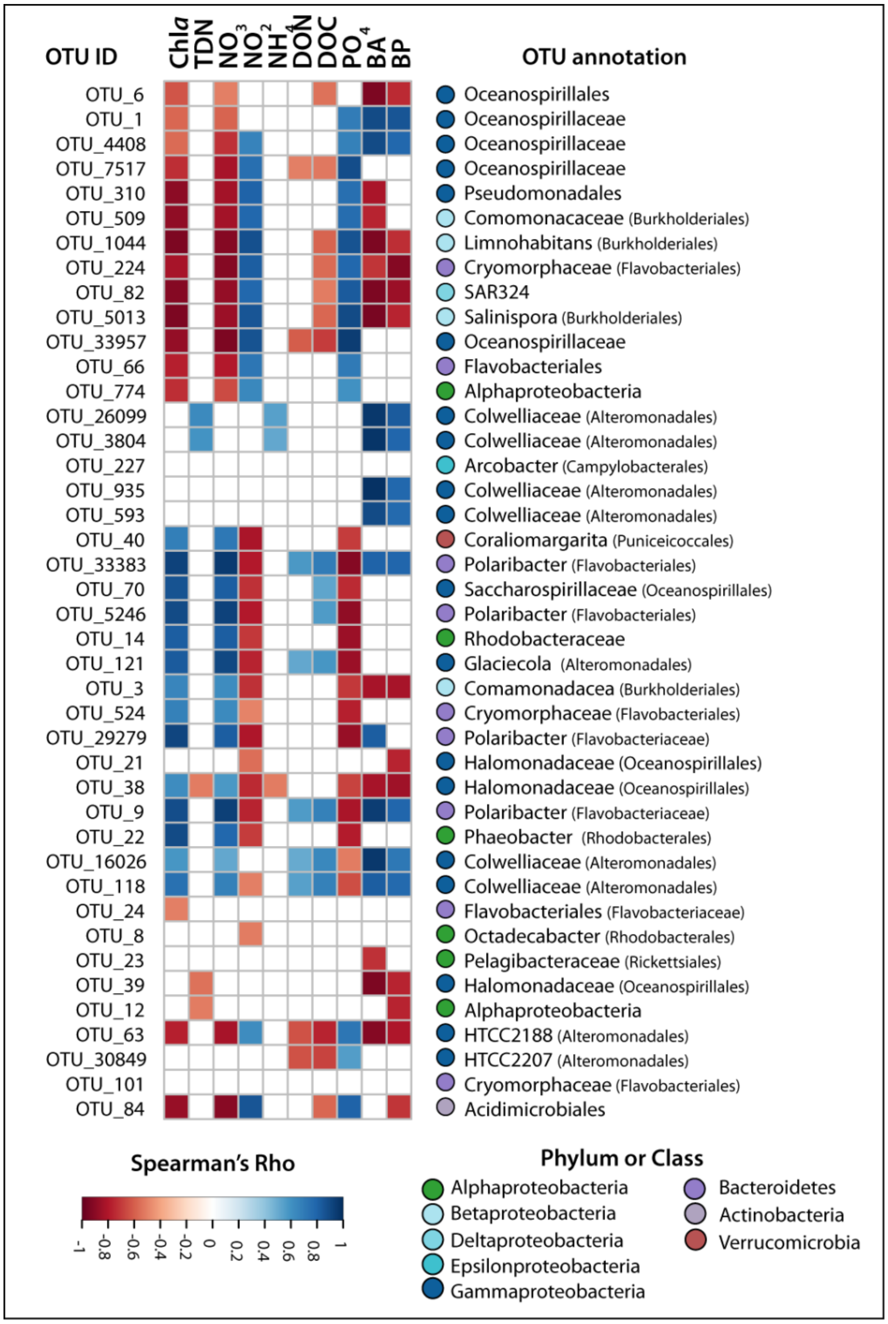

FIGURE S3 | Correlations between OTUs and biochemical parameters. Significant correlations between abundant operational taxonomic units (OTUs) that changed significantly throughout the bioassays and biochemical observations [chlorophyll (Chl a), total dissolved nitrogen (TDN), nitrate (NO_3^-), nitrite (NO_2^-), ammonium (NH_4^+), dissolved organic nitrogen (DON), dissolved organic carbon (DOC), phosphate (PO_4^{3-}), bacterial abundance (BA), and bacterial production (BP)]. Correlations were calculated using the Spearman's rank correlation statistic.
